# Hyperphosphatemic Tumoral Calcinosis: Pathogenesis, Clinical Presentation, and Challenges in Management

**DOI:** 10.3389/fendo.2020.00293

**Published:** 2020-05-08

**Authors:** Alison M. Boyce, Alisa E. Lee, Kelly L. Roszko, Rachel I. Gafni

**Affiliations:** Skeletal Disorders and Mineral Homeostasis Section, National Institutes of Dental and Craniofacial Research, National Institutes of Health, Bethesda, MD, United States

**Keywords:** fibroblast growth factor 23, phosphate, metabolic bone disease, ectopic calcification, heterotopic ossification

## Abstract

Hyperphosphatemic familial tumoral calcinosis (HFTC) is a rare and disabling disorder of fibroblast growth factor 23 (FGF23) deficiency or resistance. The disorder is manifest by hyperphosphatemia, inappropriately increased tubular reabsorption of phosphate and 1,25-dihydroxy-Vitamin D, and ectopic calcifications. HFTC has been associated with autosomal recessive pathogenic variants in: (1) the gene encoding FGF23; (2) *GALNT3*, which encodes a protein responsible for FGF23 glycosylation; and (3) *KL*, the gene encoding KLOTHO, a critical co-receptor for FGF23 signaling. An acquired autoimmune form of hyperphosphatemic tumoral calcinosis has also been reported. Periarticular tumoral calcinosis is the primary cause of disability in HFTC, leading to pain, reduced range-of-motion, and impaired physical function. Inflammatory disease is also prominent, including diaphysitis with cortical hyperostosis. Multiple treatment strategies have attempted to manage blood phosphate, reduce pain and inflammation, and address calcifications and their complications. Unfortunately, efficacy data are limited to case reports and small cohorts, and no clearly effective therapies have been identified. The purpose of this review is to provide a background on pathogenesis and clinical presentation in HFTC, discuss current approaches to clinical management, and outline critical areas of need for future research.

## Introduction

Hyperphosphatemic familial tumoral calcinosis (HFTC) is a rare and disabling disorder resulting from disturbances in FGF23-mediated phosphate regulation. Patients develop either deficiency of or resistance to FGF23, leading to hyperphosphatemia and ectopic calcifications. Inflammatory disease is also a prominent feature, including painful diaphysitis with cortical hyperostosis. Studies in patients with HFTC have led to novel insights into phosphate homeostasis, advancing our understanding of the pathophysiology of this complex disorder. In contrast, advances in clinical management have been less robust, in part due to key knowledge gaps in the natural history of HFTC and the rarity of the disease. This review will provide a background in the pathogenesis and clinical presentation in HFTC, discuss challenges in clinical management, and outline critical areas of need for future research.

## Pathogenesis

### FGF23 Function and Regulation

FGF23 is a 251-amino acid peptide secreted by osteoblasts, osteocytes (1, 2), and erythroid precursor cells of the bone marrow (3) that plays a critical role in phosphate regulation. In the proximal tubule of the kidney, FGF23 binds to the FGF receptor 1 (FGFR1) and its co-receptor KLOTHO, downregulating expression of the sodium-phosphate cotransporters NPT2a and NPT2c, which leads to phosphaturia (4). Additionally, FGF23 inhibits 1-alpha-hydroxylase and stimulates 25-vitamin D-24 hydroxylase, resulting in decreased 1,25-(OH)_2_-vitamin D (1,25D), the active form of vitamin D. Taken together, the principal actions of FGF23 lower blood phosphate. As such, elevations in blood phosphate and 1,25D stimulate FGF23 production in a classic negative feedback loop (4).

### Post-translational Processing of FGF23

FGF23 is transcribed and translated as an active, full-length protein containing a subtilisin-like proprotein convertase (SPC) site, R^176^XXR^179^/S^180^AE (5). O-glycosylation of FGF23 in the Golgi by N-Acetylgalactosaminyltransferase 3 (GalNAcT3, encoded by *GALNT3*) at Thr^178^ stabilizes the intact, active protein (6). In the absence of glycosylation, FGF23 can be cleaved by a proprotein convertase, likely furin, to its inactive C- and N- terminal fragments. Phosphorylation of Ser^180^ by the Golgi kinase FAM20C appears to inhibit glycosylation of Thr^178^ by GalNAcT3, promoting cleavage of FGF23 (5). Immunoassays may utilize antibodies targeted to either the full-length “intact FGF23,” or directed toward the C-terminal end of the molecule. Thus, “C-terminal FGF23” assays reflect a combination of both intact FGF23 and inactive C-terminal fragments.

### FGF23 in Hyperphosphatemic Familial Tumoral Calcinosis

HFTC is caused by either a deficiency of active, intact FGF23 or a defect in its signaling (Table 1). The disease is typically inherited in an autosomal recessive pattern (7). Biallelic inactivating variants in *GALNT3* cause HFTC by impairing the O-glycosylation of FGF23, leading to its cleavage and inactivation (OMIM:211900) (8, 9). Additionally, pathogenic variants in *FGF23* may lead to increased cleavage and decreased circulating intact FGF23 (OMIM:617993) (10). Biochemically, patients with *GALNT3* and *FGF23* variants demonstrate hyperphosphatemia, increased tubular reabsorption of phosphate, inappropriately normal or frankly elevated 1,25D, normal or decreased intact FGF23 and markedly elevated C-terminal FGF23 (7). Patients may also have high-normal blood calcium and low-normal parathyroid hormone levels, secondary to elevated 1,25D with increased intestinal calcium absorption. Recessive variants in the gene *KL*, which encodes the co-receptor KLOTHO, have also been shown to cause HFTC due to FGF23 resistance (OMIM:617994) (11), with elevations in both intact and C-terminal FGF23. Tumoral calcinosis has been reported in one young child with Hartsfield Syndrome due to a heterozygous inactivating variant in *FGFR1*; however, the degree of hyperphosphatemia and FGF23 resistance in that patient is unclear (12). Interestingly, autoantibodies targeting FGF23 were found to be responsible for the development of hyperphosphatemic tumoral calcinosis in a patient with no identified genetic etiology and subsequent development of type 1 diabetes (13), resulting in an acquired form of FGF23 resistance due to decreased binding of FGF23 to its receptor. Accordingly, in this patient, FGF23 levels were elevated.

**Table 1:** Causes of hyperphosphatemic tumoral calcinosis.

	**Locus**	**Gene**	**OMIM**	**Inheritance**	**iFGF23**	**cFGF23**	**Associated features**
HFTC1	2q24.3	*GALNT3*	211900	Recessive	Low/normal	High	
HFTC2	12p13.32	*FGF23*	617993	Recessive	Low/normal	High	
HFTC3	13q13.1	*KL*	617994	Recessive	High	High	Hyperparathyroidism
Autoimmune	NA	NA	NA	Acquired	High	High	Type 1 DM

The hyperphosphatemia and high-normal calcium seen with intact FGF23 deficiency or resistance leads to an increased calcium × phosphate product, which likely contributes to ectopic calcifications. Calcifications often develop in areas of inflammation, tissue hypoxia, or repetitive trauma, although it is unclear what exactly precipitates their formation.

### Mouse Models

While several models have been developed that replicate the biochemical features of HFTC, reproducing the human phenotype remains challenging. Ichikawa et al. ablated exons 2 and 3 of *Galnt3* which contain the initiation codon and part of the glycosyl transferase family 2 domain (14). As expected, this mouse developed hyperphosphatemia with decreased circulating intact FGF23, despite elevated *Fgf23* expression in the bone. This model also exhibited increased circulating C-terminal FGF23, inappropriately normal 1,25D levels, and decreased alkaline phosphatase activity; renal expression of *Slc34a1* and *Slc34a3* (the genes encoding the renal sodium-phosphate co-transporters) and *Kl* were increased. Male mice had growth retardation, infertility, and increased bone mineral density; features that have thus far not been prominently established in the human phenotype (14). Dietary phosphate restriction normalized hyperphosphatemia and the skeletal phenotype in the knockout mice, but male mice remained infertile (15). Despite biochemical abnormalities, these mice did not develop calcifications on a normal diet, but treatment with a high phosphate diet induced calcifications in approximately half of the knockout mice (16). Another HFTC mouse created by ENU mutagenesis harbors a Trp589Arg mutation in *Galnt3*, and also has hyperphosphatemia with decreased intact FGF23 levels, elevated 1,25D, and subtle periarticular calcifications (17).

## Clinical Description

There are multiple challenges in characterizing the phenotype in HFTC. The disease is exceedingly rare, and lack of high-quality prospective studies has resulted in critical knowledge gaps in its natural history. In addition, because many reported series involve multigenerational consanguinity, the presence of other autosomal recessive disorders may confound interpretation of clinical features. A clear pattern that has emerged from currently available data is that HFTC presents along a markedly broad clinical spectrum. Manifestations may vary widely, even among family members with similar biochemical profiles (18). The following sections briefly outline the clinical description and current understanding of key features in HFTC.

### Tumoral Calcinosis

**Figure 1:**
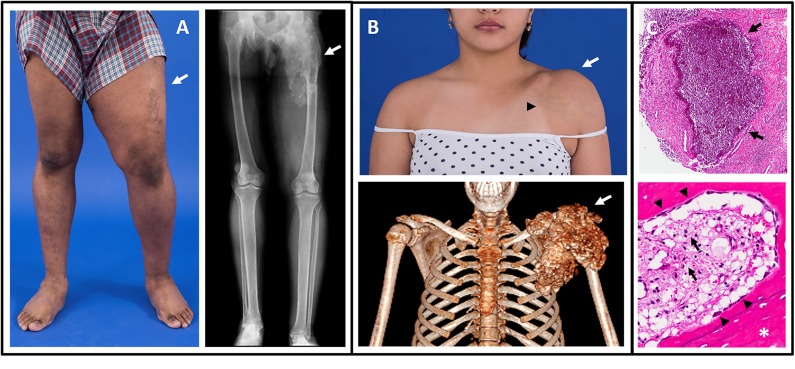
Images of tumoral calcinosis lesions. **(A)** Photograph (left panel) of a patient with swelling and decreased range-of-motion of the left lower extremity (arrow). The corresponding radiograph (right panel) shows a large area of tumoral calcinosis involving the left proximal femur. **(B)** Photograph (upper panel) of a patient with painful swelling and reduced range-of-motion of the left shoulder (white arrow). Note the overlying skin pigmentation and increased vascularity (black arrowhead). A corresponding three-dimensional computed tomography scan shows a large calcified mass involving the left shoulder (arrow). **(C)** Hematoxylin and eosin stained sections from resected tumoral calcinosis lesions. The upper panel shows a subcutaneous calcification (arrows). The lower panel shows heterotopic ossification (white asterisk) with active osteoblasts laying down new bone (black arrowheads). Note the presence of chronic inflammation with visible foamy macrophages (black arrows). These figures are original, and have not been previously published or obtained from a private database. There is no unpublished data in any of the figures.

**Figure 2:**
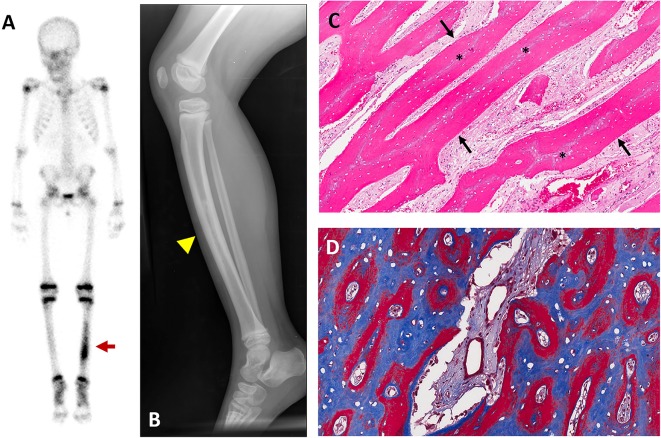
Representative images of hyperostosis. Images are from a 4-year-old girl who presented with pain and tenderness over her tibia. **(A)** Technetium-99 bone scintigraphy shows increased uptake in the bilateral tibias, greater on the left (red arrow). **(B)** Radiograph of the left tibia shows thickened cortices with patchy radio-opacities in the medullary canal (yellow arrowhead). **(C)** Hematoxylin and Eosin stained section of periosteal bone from a tibial biopsy shows sheets of mineralized lamellar bone (arrows) replacing central areas of woven bone (asterisks). **(D)** Goldner trichrome staining in an undecalcified section reveals mature lamellar bone in blue and woven bone in red. These figures are original, and have not been previously published or obtained from a private database. There is no unpublished data in any of the figures.

Ectopic calcifications in the skin and subcutaneous tissue are a classic and potentially morbid feature of HFTC (Figures 1, 2). Lesions consist of hydroxyapatite and/or calcium carbonate (19, 20) (Figure 1C), and typically occur in peri-articular locations that are exposed to repeated pressure or trauma (7). The lateral hips are the most frequently affected site, but a wide range of areas may be involved, including the elbows, shoulders, hands, Achilles tendons, and others (11, 18). Calcifications typically onset during the first two decades of life, and have been reported in children as young as 6 weeks (21). Once present, lesions may grow slowly over time. Patients present along a broad spectrum, ranging from no involvement to lesions that are large, painful, and debilitating (18, 22). Calcifications that occur around joint spaces can impair mobility and physical function. In severe cases, calcific tumors perforate the skin and drain liquid hydroxyapatite, which may be confused with purulent drainage. Ulceration may also be accompanied by recurrent secondary infections.

On radiographs tumoral calcinosis appears as heterogeneous calcified masses (18) (Figure 1A). Computed tomography scans offer detailed anatomical information and are thus a sensitive test to both detect lesions and monitor their progression (Figure 1B); however, clinicians should be judicious in balancing the benefits of imaging with the risks of radiation exposure.

### Hyperostosis

Hyperostosis is a unique and poorly understood characteristic of HFTC (Figure 2). Patients present with pain and tenderness overlying the diaphyseal regions of long bones, often accompanied by edema, erythema, and warmth (18, 22, 23). The tibias are most commonly affected, but multiple sites may be involved, including the ulnas, radii, and metacarpals (24, 25). Symptoms may onset acutely with a variable duration and may recur episodically, leading to significant pain and functional impairment. A misdiagnosis of osteomyelitis is frequent, particularly if hyperostosis is the initial presenting feature.

Radiographs often show pronounced periosteal reaction with hypermineralized cortical bone and patchy sclerotic areas involving the medullary canal (Figure 2B). Lesions are typically active on nuclear medicine scan (Figure 2A). Biopsies typically reveal areas of reactive bone with fibroblastic stroma, which are infiltrated with inflammatory polymorphonuclear cells and lymphocytes (18, 22) (Figures 2C,D).

### Inflammatory Disease

Patients may exhibit clinical signs of systemic inflammation, including recurrent fevers, fatigue, anemia, and polyarthritis, often accompanied by increased serum levels of C-reactive protein and/or erythrocyte sedimentation rate (18, 26–28). The etiology of inflammation in HFTC is unknown but is speculated to be related to macrophagic engulfment of hydroxyapatite crystals in calcific lesions (18) (Figure 1C). Preliminary support for this concept is provided by the clinical literature: (1) all reported patients with systemic inflammation also had large calcifications (18, 26–28), and (2) two patients who had resolution of calcified lesions after treatment also had concomitant improvement in serum inflammatory markers and symptoms of inflammation (18, 26). Further investigation is needed to define the relationship between tumoral calcinosis lesions and inflammation, as well as the clinical sequelae of chronic inflammation in patients with HFTC.

### Ocular Involvement

Ocular manifestations are an uncommon but distinct feature of HFTC. Calcifications may involve the eyelids and/or conjunctiva, which may present with eye itching and irritation (29–32). Corneal calcifications have led to band keratopathy in several patients (31, 32). Retinal angioid streaks have been reported (33, 34), likely arising from calcification of the elastin-rich membrane between the retina and choriocapillaris (35). One patient with HFTC developed sudden vision loss as a result of a subretinal hemorrhage from choroidal neovascularization of a retinal angioid streak (36).

### Other Calcifications

**Figure 3:**
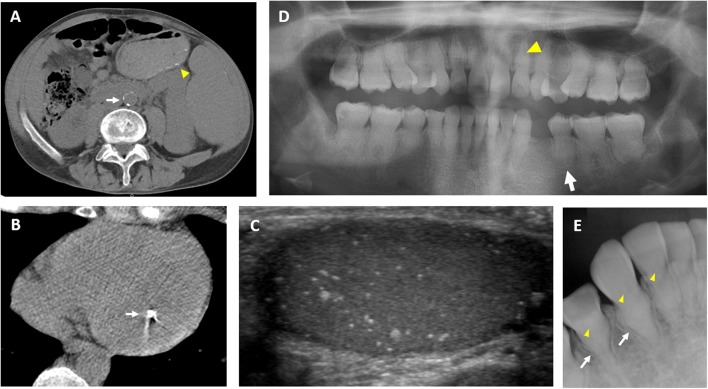
Clinical images of extraskeletal features in hyperphosphatemic tumoral calcinosis. **(A)** Computed tomography scan from a 36-year-old man demonstrates calcification in the abdominal aorta (white arrow). Small submucosal bowel calcifications are also visible (yellow arrowhead). **(B)** Computed tomography scan of the heart in a 29-year-old man demonstrates a calcified lesion in the papillary muscle (arrow). **(C)** Testicular ultrasound from a 32-year-old man showing diffuse microlithiasis. **(D)** Panoramic dental radiograph from an 18-year-old woman shows teeth with short, bulbous roots (white arrow) and obliteration of dental pulp (yellow arrowhead). **(E)** Periapical dental radiograph from a 12-year-old girl shows thistle-shaped root (white arrows) and obliteration of dental pulp (yellow arrowheads). These figures are original, and have not been previously published or obtained from a private database. There is no unpublished data in any of the figures.

Calcifications may affect small and large vessels in various locations, including the aorta, iliacs, carotids, cerebral vasculature, and others (11, 18, 32, 37, 38) (Figure 3A). Patients may present with signs of peripheral vascular disease, including pain and diminished peripheral pulses, which in severe cases have necessitated amputations (38). Cardiac calcifications may include the coronary vessels or muscular structures (18) (Figure 3B). Coronary artery calcifications and systemic inflammation are both established risk factors for cardiac disease; prospective studies are needed to evaluate cardiac outcomes in patients with HFTC and determine therapeutic strategies.

Calcifications may occur in a variety of other extracutaneous tissues, including the dura (22), tongue (33), and submucosal gastrointestinal tract (18) (Figure 3A). Testicular microlithiasis was associated with decreased sperm production in one patient (25) (Figure 3C). Nephrocalcinosis has been reported, which was associated with decreased renal function in one patient (30, 39).

### Dental Involvement

Dental pathology is one of the most penetrant features of HFTC (7). Prominent dental abnormalities such as shortening of roots and partial obliteration of dental pulp are commonly seen on panoramic and periapical radiographs (Figures 3D,E). Findings are similar to dentin dysplasia, including short bulbous teeth with abnormal calcifications (18, 40–43). While generalized short roots can be seen in numerous syndromes, teeth of patients with HFTC reveal a unique thistle-shaped root with gross enlargement in the coronal third of the root and acutely tapering apical third (43–45). Abnormal curvature of the dental roots, known as dilacerations, is seen in some patients (18, 45). Additionally, varying degrees of obliteration of the pulp chamber and root canal is commonly observed (7, 18, 40–42). The pulp, normally an unmineralized oral tissue composed of vascular, nervous, and connective tissue, are often obstructed with pulp stones in HFTC (7, 18). Complete obliteration of the pulp space is also observed in patients (46). The presence of abnormal calcification in the pulp space can hinder root canal therapies (47). Currently, the etiology of dental pulp obliteration and root dysmorphology in HFTC is unknown. It is important for dentists to be aware of the unique presentation of pulpal obliteration and thistle-shaped roots because the dental radiographic findings can be the first sign of the disease in patients without other systemic manifestations (2, 8, 11).

Clinically, the tooth morphology of patients with HFTC appear normal in color, size, and shape (42, 48). Most studies have described a healthy oral mucosa in patients with HFTC (43, 44, 49). Although enamel hypoplasia has been observed in several case reports, enamel is rarely affected in most patients (50). Bilateral maxillary and mandibular tori have been reported in some patients, which can interfere with tongue movement and speech (42, 51). The rare presence of enamel abnormalities can increase caries risk in HFTC; more commonly found pulp calcifications do not allow endodontic treatment of caries (50). Thus, application of dental sealants for caries prevention is suggested as a better treatment option.

### Other Manifestations

Phenotyping studies are likely to uncover additional clinical manifestations in patients with HFTC, particularly features that are less prevalent or more subtle in their presentation. Much interest has been paid to “off-target” effects of FGF23 in other metabolic bone disorders, such as X-linked hypophosphatemia and chronic renal insufficiency, where excess FGF23 has been associated with varying effects on the cardiovascular, gastrointestinal, immune, and central nervous systems (4). Emerging evidence demonstrates that FGF23 processing is affected by inflammation and iron metabolism, which may be relevant for patients with HFTC who may demonstrate both chronic inflammation and anemia (52). Studies are needed to determine if these and other “off-target” effects affect patients with different forms of HFTC.

## Treatment

Pathogenic variants in *GALNT3* or *FGF23* result in functional FGF23 deficiency, thus hormone replacement therapy with FGF23 would be the ideal treatment for most causes of HFTC. Until this becomes available, current interventions focus on managing blood phosphate, reducing pain and inflammation, and addressing calcifications and their complications. Unfortunately, efficacy data are limited to case reports and small cohorts. In addition, the lack of longitudinal studies has led to knowledge gaps in the natural history of HFTC, further confounding interpretation of treatment efficacies.

### Phosphate-lowering Therapies

A low phosphate diet is recommended for patients with HFTC, although there is limited evidence that this alone is sufficient (53). As phosphate is abundant in many foods, particularly those high in protein such as dairy, nuts, and meat, consultation with a dietician may be necessary to assist with meal planning. The United States Recommended Daily Allowance for phosphate ranges from 500 to 1,250 mg/day in children and adolescents and 700 mg/day in adults (54). However, because of the high protein diet in the United States, most individuals consume at least twice the Recommended Daily Allowance (55). Similar to patients with hyperphosphatemia secondary to renal insufficiency, patients with HFTC are typically recommended to restrict phosphate intake to 600–800 mg/day (less in young children), which is difficult for many to achieve (56).

Medications that inhibit intestinal absorption of dietary phosphate have been tried in patients with HFTC with varying success, including sevelamer, lanthanum, and aluminum hydroxide (7, 18, 22, 26, 50, 57–59). To be effective, phosphate binders should be given with all meals and snacks. Common side effects include constipation, nausea, and abdominal pain; in rare cases, intestinal obstruction or perforation can occur. Aluminum toxicity is unlikely as renal function is usually normal in patients with HFTC, however, this potential complication should always be considered when choosing this therapy. Lanthanum is a soft metal which is radiopaque and, while benign, can be mistaken for intestinal foreign bodies on abdominal radiographs (60). Calcium salts, which are often used to lower blood phosphate in other disorders, should be avoided in patients with HFTC, as these could potentially increase the calcium-phosphate product and worsen calcifications. As HFTC is associated with high 1,25D, vitamin D supplements should never be administered to these patients, even in the face of a low 25-OH-vitamin D level.

Acetazolamide, a carbonic anhydrase inhibitor which induces a proximal tubular acidosis, is commonly used in HFTC. Efficacy is variable, with some reporting a decrease in blood phosphate, tubular reabsorption of phosphate, and calcific tumors with others reporting no obvious benefit (18, 26, 45, 58, 59, 61). It has been suggested that its mode of action on calcifications is not through promoting renal phosphate excretion, but rather by increasing calcium-phosphate solubility through lowered serum pH (61). Serum bicarbonate should be monitored periodically; while the lower acceptable limit of bicarbonate is not known, a level of 18–20 mmol/L is likely to be tolerated without significant complications.

Probenecid is a uricosuric agent that also increases renal phosphate excretion and has been tried in tumoral calcinosis (18, 22, 59). It should be used with caution when co-administered with other medications, as probenecid can increase the half-life of many drugs (e.g., certain antibiotics), leading to potential toxicity. Nicotinamide, a drug which downregulates sodium-phosphate co-transporters in the kidney and intestine, was shown to decrease progression of calcifications in *Galnt3* knock-out mice without an effect on serum phosphate levels (62). Brief treatments with niacinamide and nicotinamide have been tried in a small number of patients with HFTC (18, 45, 63), but there are no available long-term data.

### Anti-inflammatory Therapies

In patients with significant inflammatory disease, often evidenced by erythema, lesional warmth, intermittent fevers, elevated erythrocyte sedimentation rate and/or c-reactive protein, anti-inflammatory medications may be useful. Non-steroidal anti-inflammatory drugs (NSAIDS) and glucocorticoids have been reported to improve symptomatic hyperostosis (11, 27). Blockade of interleukin-1 (IL-1) action with anakinra, an IL-1 receptor antagonist, and canakinumab, a monoclonal antibody against IL-1β, have been shown to reduce inflammation and pain and improve quality of life in a small number of patients (18, 64); inflammatory calcifications resolved in one (18).

### Anti-mineralization Therapies

Sodium thiosulfate, approved for use as an antidote to cyanide poisoning, also appears to have anti-mineralization properties due to several proposed, yet unconfirmed, mechanisms such as increasing the solubility and excretion of calcium through chelation, and acting as an antioxidant to reduce inflammation (65). As a result, intravenous, oral, topical, and intralesional sodium thiosulfate has been studied in a variety of calcific disorders including nephrolithiasis (66), autoimmune calcinosis cutis (67), dermatomyositis-related tumoral calcinosis (68, 69), and calcific uremic arteriopathy (70). In HFTC, topical application of sodium thiosulfate decreased calcifications in three patients after many months of therapy (24); intravenous and intralesional applications have not been reported in HFTC.

### Surgical Resection

Surgical outcomes are highly variable, with some patients experiencing recurrence of lesions or poor wound healing (18, 22, 53). Given the risks, surgery is often reserved for those with severe lesions affecting activities of daily living or chronic drainage and infections.

### Physical and Occupational Therapy

As joints are most commonly affected by tumoral calcinosis, patients often have reduced range of motion and/or pain that can severely impair activities of daily living, including walking, eating, and routine hygiene. Consultation with physiatrists and therapists are an important and often underutilized resource to ensure that patients are taught management strategies and provided with adaptive devices.

### Other Therapies

Given the inconsistent efficacy of the treatments listed above, there have been attempts to manage HFTC with calcium-channel blockers, bisphosphonates, ketoconozale, methotrexate, parathyroid hormone, and TNF-blockade, most without demonstrable improvement. Calcitonin appeared to lower phosphate and stabilize calcifications in one case (71). In a patient with ocular compromise due to angioid streaks, intravitreal injections of ranibizumab (an antibody to vascular endothelial growth factor) improved visual acuity (36). Immunomodulatory therapy has not been tried in the one known patient with autoimmune hyperphosphatemic tumoral calcinosis, because he has responded generally well to conventional therapy. Patients with vascular calcifications require evaluation and management by a vascular specialist and/or cardiologist. Consultation with a pain specialist may be needed in severe cases of HFTC. As with other chronic pain conditions, involvement of a mental health specialist may also be beneficial.

## Future Directions

Until gene therapy become routine practice, hormone replacement therapy with recombinant or synthetic FGF23 would be the optimal treatment for patients with HFTC due to *GALNT3* or *FGF23* variants. Other approaches, particularly for autoimmune and FGF23-resistant forms of tumoral calcinosis, include targeted therapies that inhibit renal phosphate reabsorption. For example, a recent study using an oral inhibitor of a renal sodium-phosphate co-transporter increased phosphate excretion and reduced serum phosphate in a murine model of chronic kidney disease (72). Whether this class of drugs will be effective in HFTC remains to be seen.

There are multiple challenges in designing interventional trials in HFTC. The development of calcified lesions is by nature an intermittent process, therefore prospective observational studies are critical to define the natural history of the disease, and to understand the phenotypic variability between individuals. Clinically relevant surrogate endpoints are another key area of need for future research. Phosphate levels and inflammatory markers are intuitive potential biomarkers, however more robust studies correlating these levels with clinical outcomes are needed. Investigation is also needed to define optimal imaging strategies to detect, monitor, and predict lesion development.

## Conclusion

Investigation in HFTC has greatly expanded our knowledge of phosphate homeostasis, leading to novel insights into FGF23 signaling. HFTC may result from dysregulation in multiple pathways, resulting in states of FGF23 deficiency and/or resistance. In all forms of HFTC, periarticular tumoral calcinosis is the primary cause of disability. In contrast to recent advances in understanding of HFTC pathophysiology, the development of clinical management approaches has lagged. Multiple treatment strategies have attempted to manage blood phosphate and reduce pain and inflammation; however, efficacy data are limited to case reports and small cohorts, and no clearly effective therapies have been identified. There is a critical need for longitudinal natural history studies to identify therapeutic targets and surrogate endpoints to improve outcomes for patients with HFTC. FGF23 replacement is an intuitive potential treatment strategy for most patients and should be considered a high priority for future research.

## Ethics Statement

Written informed consent was obtained from the individual(s) AND/OR minors' legal guardian for the publication of any potentially identifiable images or data included in this article.

## Author Contributions

All authors contributed to manuscript preparation, revision, read and approved the submitted version.

## Conflict of Interest

The authors declare that the research was conducted in the absence of any commercial or financial relationships that could be construed as a potential conflict of interest. The handling Editor declared a past co-authorship with one of the authors AB.
